# Surgical margin clearance and extended chemotherapy defines survival for synchronous oligometastatic liver lesions of the ductal adenocarcinoma of the pancreas

**DOI:** 10.1007/s10147-021-01961-5

**Published:** 2021-06-16

**Authors:** S. A. Safi, G. Fluegen, A. Rehders, L. Haeberle, S. Fung, V. Keitel, A. Krieg, W. T. Knoefel, N. Lehwald-Tywuschik

**Affiliations:** 1 Department of Surgery (A), Medical Faculty, Heinrich-Heine-University and University Hospital Dusseldorf, Moorenstr. 5, 40225 Dusseldorf, Germany; 2 Institute of Pathology, Medical Faculty, Heinrich-Heine-University and University Hospital Dusseldorf, Dusseldorf, Germany; 3 Department of Gastroenterology, Hepatology and Infectious Diseases, Medical Faculty, Heinrich-Heine-University and University Hospital Dusseldorf, Dusseldorf, Germany

**Keywords:** PDAC, Ductal adenocarcinoma of the pancreas, Hepatic metastases, Oligometastatic, Survival outcome

## Abstract

**Background:**

The role of surgery for circumscribed synchronous hepatic lesions of the pancreatic ductal adenocarcinoma (PDAC) remains controversial. Thus, the aim of our study was to compare survival outcome (OS) after surgery of patients with hepatic metastases (M1surg) to patients with only localized disease.

**Methods:**

Correlation analysis of clinicopathological data and OS after resection of M1surg patients and patients with localized PDACs (M0) was performed. Patients were included for survival analysis only if a complete staging including perineural, venous and lymphatic invasion was available.

**Results:**

Out of the study collective, 35 patients received extended surgery (M1surg), whereas 131 patients received standardized surgery for localized disease (M0). Length of hospitalization and mortality was similar in both groups. FOLFIRNOX as an adjuvant treatment regime was administered in ~ 23 and ~ 8% of M1surg and M0 patients, respectively. In subgroup analysis of R0 resected patients and in multivariate analysis of the total cohort, there was no difference in overall survival between both groups. Only the resection status (R1 vs R0) and venous invasion (V1) were identified as independent prognostic factors. Site of recurrence in R0 resected M1surg patients and in M0 patients were homogenously distributed.

**Conclusion:**

This is the first study demonstrating a survival benefit after extended surgery for synchronously hepatic-metastasized PDACs. We found no difference in survival outcome of metastasized patients when compared to patients with localized disease. FOLFIRINOX as an adjuvant treatment regime for resected M1surg presumably is worthwhile. Larger multicenter studies are still needed to validate our results.

**Supplementary Information:**

The online version contains supplementary material available at 10.1007/s10147-021-01961-5.

## Background

The ductal adenocarcinoma of the pancreas (PDAC) has a poor prognosis with a median overall survival of ~ 6 months and is estimated to become the second leading cause of cancer-related death in the United States and also in Germany by 2030 [1, 2]. To date, the only curative therapy remains the margin-negative oncological resection with an adjuvant treatment regime starting within 6 weeks of the operation [3, 4]. Because oncological advances for PDAC have been slow and poor, the 5 year overall survival rate did not change over the past decade and remains under 10% [5].

The PDAC metastasizes primarily the peritoneum, the liver and to the lungs [6]. At diagnosis of PDAC, 50% of patients have already metastasized synchronously and further 30% presented with locally advanced disease, which is not suitable for surgery. Thus, only 20% of the patients with a PDAC received curative-intended surgery. Therefore, it is still regarded as one of the most lethal cancers indicated by a very high mortality-to-incidence ratio [5, 7].

Palliative intended therapy or chemotherapy is the standard of care for patients with metastasized or locally advanced PDACs [8, 9]. To date, however, no standardized surgical treatment exists for patients with synchronous or metachronous oligometastatic disease. Therefore, in current clinical practice, unlike in other malignancies, synchronous metastasectomy of PDAC has rarely been performed. In these patients, neoadjuvant chemotherapy with subsequent resection and ablative technologies are possible treatment options for metastasized PDAC. Hence, therapeutic regimes, such as FOLFIRINOX (folinic acid, fluorouracil, irinotecan, oxaliplatin) or gemcitabine and nab-paclitaxel, have very recently been established as neoadjuvant or primary treatment options [9, 10]. To date, it is unclear which patient group might benefit from such an individual approach of neoadjuvant therapy followed by radical tumor resection. Moreover, it is unclear whether chemotherapy-naive patients with small tumor burdens, patients with a stable disease, or patients with tumor regression after neoadjuvant therapy would benefit from a multimodal approach.

The aim of our study was to analyze patients who received extended surgery in our department for synchronously hepatic-metastasized ductal adenocarcinomas of the pancreas (M1surg) and to compare those to two control groups: patients after multimodal therapy for localized disease (M0) and patients who received palliative intended therapy for metastasized disease (M1pall).

## Methods

### Patient selection and clinicopathological data

Patients with ductal adenocarcinoma of the pancreas who consecutively received surgery or palliative therapy between Sep 2006 and Dec 2019 at the Heinrich Heine University Hospital of Dusseldorf were included in the study. Exclusion criteria were patients with (1) malignancies of the pancreas other than ductal adenocarcinoma, (2) in whom the TNM staging did not include information about lymphatic, perineural and venous invasion (Lx, Pnx, Vx), (3) in patients who were lost to follow-up, (4) in patients who received palliative intended therapy other than for isolated resectable hepatic metastases, (5) in patients who succumbed within the 30 day of surgery and (6) in which intraoperatively a routine liver sonography was not documented. Cut off point during follow-up was 60 months. Clinical data of these consecutively treated patients collected from patient’s medical records were compiled into an Excel-file database and analyzed retrospectively.

Oligometastastic disease was defined as resectable hepatic metastases isolated in one hepatic lobe, accessible only via an atypical resection, and independent on size and amount of metastases. Patients who received palliative intended therapy were included only if information about the number, size and location of the hepatic metastases were available. This data was compared to patients with extended surgery for metastasized disease. Information of the TNM staging system (size of tumor/involvement of adjacent arteries, lymph node status, and status on distant metastasis), along with grading, perineural invasion, lymphatic and venous invasion was retrospectively collected from the original histopathological reports for each patient. The TNM staging system, if applicable, was updated to the eighth edition of the UICC TNM classification of malignant tumors [17]. Stated R-status of each patient was dependent on the pancreatic/logoregional as well as hepatic specimens. Size by greatest diameter measured pathologically, and the location and number of hepatic metastases were re-assessed from the pathological reports and radiographic imaging. Clinico-pathological data (gender, age at the time of surgery, overall survival (OS) and results of follow-up examinations including time of diagnosis of metastases and sight of metastases) were retrieved. If the follow-up examinations were performed at our institution, irrespective of the treatment constellation, computed tomography of the thorax and abdomen was performed every 3 months for the first 2 years, followed by every 6 months thereafter. Patients with suspicious metachronous masses were discussed in the tumor board for further therapy. If follow-up procedures were performed at other institutions, survival records of patients were gathered from the legal registration office.

The analysis was performed in conformity to the Declaration of Helsinki and to good clinical practice. Furthermore, the study war approved by the Institutional Review Board (IRB) of the Ethics Committee, Heinrich Heine University Dusseldorf (IRB-no. 2019–473-2).

### Statistical analysis

The Wilcoxon test was used to analyze differences in clinicopathological data between the three subgroups. The Mann–Whitney *U* test was used to examine numerical data and to correlate between clinic-pathological variables. For categorical data, the chi-square test was applied. The overall survival (OS) was determined as the period from the date of surgery until the date of death of any cause, or the last follow-up. Disease-free survival (DFS) described the period from the date of surgery until the date of diagnosed metachronous metastases or local recurrence. To perform the above mentioned correlation and survival analysis in one single study cohort, patients who succumbed during the first 30-postoperative days were removed from analysis and were only presented for correlation of mortality rate. Kaplan–Meier curves were generated and analyzed using the log-rank (Mantel Cox) test, and hazard ratios (HRs) with 95% confidence intervals (CIs) were estimated. For multivariate survival analysis, all variables were included into a logistic regression analysis. Analyses were performed using SPSS® statistics for Windows (version 25.0; SPSS, Inc., Chicago, IL, USA). *p* < 0.05 was considered to indicate a statistically significant difference.

## Results

From a total cohort of 346 patients who scheduled surgery for PDAC with curative intend, regardless of tumor stage, 195 patients met our pre-defined inclusion criteria for the analysis of synchronous-metastasized PDAC and received oncologic surgery (pancreatic surgery with/without hepatic metastasectomy) in our hospital. 38 patients met the inclusion criteria of oligometastatic disease to the liver (group M1surg). In the same period, 143 consecutive patients were scheduled for surgery for localized disease (group M0). Fifteen patients succumbed during the first 30-postoperative days (Clavien-Dindo V 7.7%), which is in-line with published mortality rates [11]. These were excluded from the study, which now includes 35 M1surg and 131 M0 patients in the study group (Table 1). There was no statistical difference in mortality rates between groups M0 and M1surg (Clavien–Dindo V 7.9% for M1surg and Clavien–Dindo V 8.3% for M0, fisher exact test *p* = 0.450). Further 14 patients with oligometastatic disease to the liver and a similar ECOG performance status to group M0 and M1surg (group M1pall), who did not agree on an extended surgical approach, were treated with a palliative intended chemotherapy according to national guidelines [12]. None of the palliative treated patients succumbed during the first 30 chemotherapeutic days. In all 180 patients, an intraoperative ultrasound of the liver was performed and documented for further analysis.

**Table 1:** Demographic table of all 180 studied patients divided into three groups: M0, M1surg and M1pall

	M0 *n* = 131		M1 surg *n* = 35		M1 pall *n* = 14	
Age in years						
Median (range)	69 (17–95)		67 (45–80)		71.5 (51–87)	
Gender	*n*	%	*n*	%	*n*	%
Male	80	38.9	20	42.9	7	50
Female	51	61.1	15	57.1	7	50
Tumor location						
Head	119	90.8	27	77.1	13	92.9
Tail	12	9.2	8	22.9	1	7.1
T-stage						
T1	8	6.1	4	11.4	–	–
T2	78	59.5	11	31.4	–	–
T3	44	33.6	18	51.4	–	–
T4	1	0.8	2	5.7	–	–
N-stage						
N0	27	20.6	7	20.0	–	–
N1	99	75.6	27	77.1	–	–
N2	5	3.8	1	2.9	–	–
Grading						
G1/G2	81	61.8	17	48.6	12	85.7
G3	50	38.2	17	48.6	2	14.3
Missing	–	–	1	2.9	–	–
Pn						
Pn0	30	22.9	11	31.4	–	–
Pn1	101	77.1	24	68.6	–	–
Missing	–	–	–	–	–	–
L						
L0	74	56.5	18	51.4	–	–
L1	57	43.5	17	48.6	–	–
Missing	–	–	–	–	–	–
V						
V0	96	73.3	23	65.7	–	–
V1	35	26.7	12	34.3	–	–
Missing	–	–	–	–	–	–
R-status						
R0	111	84.7	17	48.6	–	–
R1	20	15.3	18	51.4	–	–

*surg* surgical, *pall* palliative, *Pn* perineural invasion, *L* lymphatic invasion, *V* venous invasion

The median age of all 180 patients at the time of surgery was 68 years (range 17–95 years). Our collective consisted of 107 males (59.4%) and 73 females (40.6%) and did not show any differences within the three groups. In 159 patients, the PDAC was located in the pancreatic head. In further 21 patients, the tumor originated from the pancreatic tail (Table 1). In our total cohort of patients, the mean follow-up period was 36.0 months (95% CI 29.1–42.9 months).

The median hospital stay for surgically resected patients with localized disease (M0) and for patients who received extended surgery for metastasized disease (M1surg) was 22 days (range 9–262 days) and 21 days (range 10–88 days) respectively (*p* = 0.503) (Table 2). In group M1pall, the median hospital stay was significantly shorter compared to both other groups (median days: 11 days, range 5–15 days) (Table 2).

**Table 2:** Correlation analysis of subgroups (M0, M1 surg and M1 pall) and clinicopathological variables in PDAC

	M1 surg vs M0 (*p *value)	M1 surg vs M1 pall (*p *value)	M0 vs M1 pall (*p *value)
Tumor location	0.039	0.563	0.347
Age	0.132	0.031	0.173
Gender	0.701	0.703	0.833
T-stage	0.014	–	–
N-stage	0.957	–	–
Grading	0.428	0.087	0.040
Pn	0.377	–	–
L	0.702	–	–
V	0.402	–	–
R-status	< 0.001	–	–
Morbidity	0.665	0.003	0.001
Hospital stay (days)	0.503	0.001	0.002

Pearson test was used to test for statistical significance. *p* value ≤ 0.05 indicates significance

Hospitality length was significantly shorter in M1pall patients

*surg* surgical, *pall* palliative, *Pn* perineural invasion, *L* lymphatic invasion, *V* venous invasion

### Correlation analyses of clinicopathological variables

Of all analyzed clinicopathological variables, location of the PDAC (head vs. tail), T-stage and R-status were heterogeneously distributed between patients who received curative-intended surgery for localized and metastasized disease respectively (M0 vs M1surg) (Table 2). Thus, a larger tumor size correlated with synchronous hepatic metastases. Of the 18 M1surg patients with R1 resections, in 10 patients (55.6%) margin clearance could not be achieved at site of liver metastasectomy. Thus, the peripancreatic resection status was of no statistical difference between group M0 and M1surg (peripancreatic R0 status in M0 = 84.7 vs 77.1% in M1surg, *p* = 0.312). Furthermore, the distribution of resection status (R1 vs R0) was independent on the number, size and sight of liver metastases in group M1surg (Supplemental Table i).

A correlation analysis of pathological data in the group with palliative intended therapy was not performed due to incomplete pathological staging for the primary tumor (Table 1 and 2). As evident in computed tomography, documented intraoperative sonography and histopathological reports, the size and number of hepatic metastases were homogeneously distributed between M1pall and M1surg patients (Table 3). In median, one metastasis (range 1–4) was resected in patients with synchronously metastasized PDAC, and diagnosed via surgical exploration in group M1pall (range 1–2). In all 35 patients, atypical parenchyma sparing liver resections were performed. In 27 (51.9%) surgically treated patients and in nine (64.5%) patients with palliative intended treatment, the metastases were located in the left hepatic lobe. In correlation analysis, there was no significant difference in number, size and site of metastases between each group (Table 3).

**Table 3:** Correlation analysis of metastatic configuration of the two subgroups (M1 surg and M1 pall)

	M1 surg *n* = 35	M1 pall *n* = 14	Fisher-exact test *p* value
Number of metastases			0.111
Single lesion	21	12	
2 lesions	8	2	
3 lesions	3	0	
4 lesions	3		
Size of metastases			0.246
< 2 cm	26	13	
≥ 2 cm	9	1	
Location of metastases			0.426
Left lobe	19	9	
Right lobe	16	5	

Amount, size and location of metastases were homogenously distributed between group M1 surg and M1 pall

*surg* surgical, *pall* palliative

^*^p value ≤ 0.05 indicates significance

### Survival analysis

Out of the 180 patients, 117 patients (65.0%) died during the follow-up period. The median OS of all 180 patients was 15.1 months (95% CI 10.4–19.8 months). Out of patients who received curative-intended therapy (M0 and M1surg, *n* = 166), 90.9% of the patients received a multimodal therapy (Supplemental Table ii). In group M0, 80 patients (61.1%) were given gemcitabine as monotherapy, whereas 35 patients (26.7%) received a combination therapy with paclitaxel. Only five patients (3.8%) were given FOLFIRINOX as a standardized adjuvant treatment regime. None of the M0 patients received neoadjuvant treatment. In the M1surg group, 15 patients received an adjuvant gemcitabine therapy (42.8%), while eight patients received FOLFIRINOX (22.8%) (four perioperative and four postoperative) and two patients received an adjuvant gemcitabine multidrug regime with either erlotinib or paclitaxel (5.7%). Further five patients entered the HEAT study and received adjuvant radiochemotherapy (14.2%). The distribution of chemotherapeutic regimes was heterogeneous between group M0 and M1surg (*p* < 0.001) (Supplemental Table ii).

### Overall survival

**Fig. 1:**
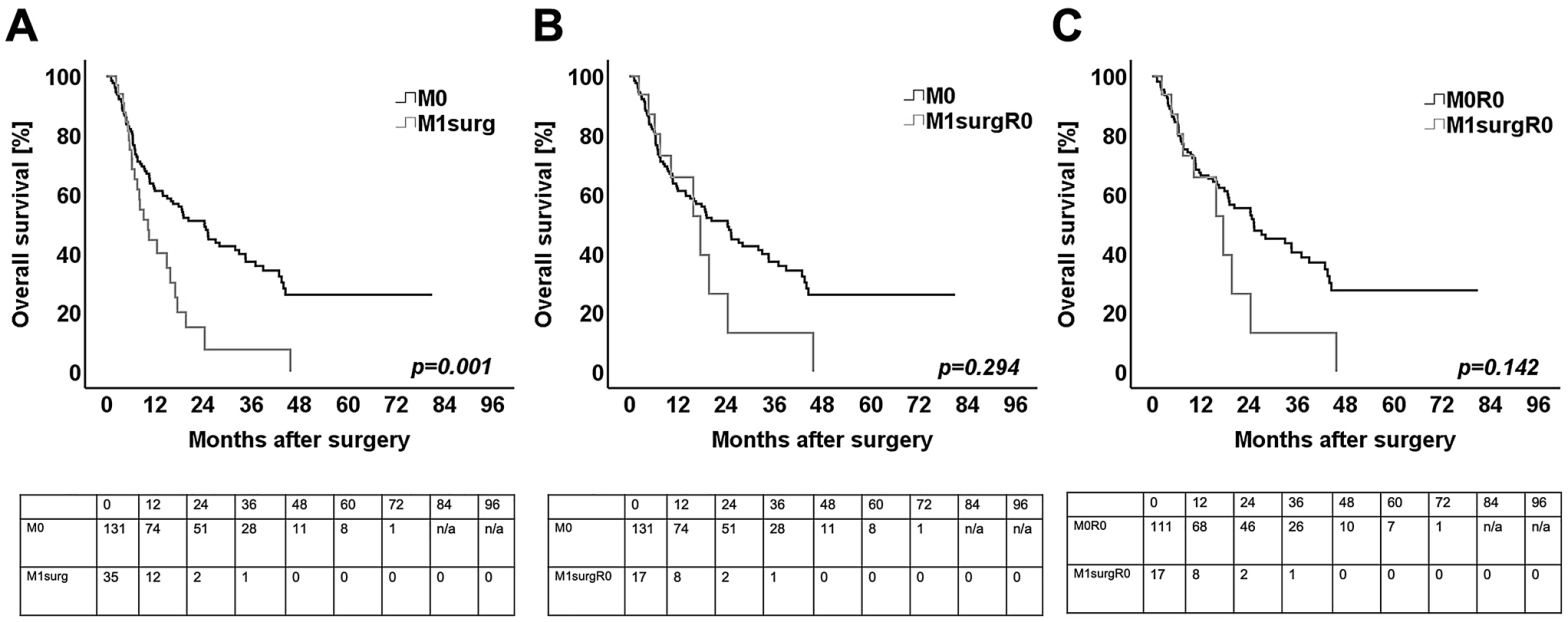
Kaplan Meier survival curves for **A** overall survival of patients without synchronous metastases (M0, *n* = 131) in correlation to patients with extended surgery (M1surg, *n* = 35) **B** overall survival of patients without synchronous metastases (M0, *n* = 131) in correlation to patients after margin-negative extended surgery (M1surgR0, *n* = 17) **C** overall survival of patients after margin-negative resections without synchronous metastases (M0R0, *n* = 111, Table 5) in correlation to patients after margin-negative extended surgery (M1surgR0, *n* = 17, Table 5). Log rank test was used to test for significance. *p* value ≤ 0.05 indicates significance

Univariate survival analysis was performed for the total cohort. In the univariate analysis of all 166 surgically resected patients (M0 and M1surg), patients with: higher median age, PDACs of the pancreas tail, surgically resected synchronous hepatic metastases, higher tumor grading, positive venous infiltration, positive resection margins and single drug chemotherapy had a significantly worse overall survival (Table 4). Thus, patients who received resection of the primary PDAC with synchronous liver metastases had a median OS of 10.3 months (95% CI 7.2–13.4 months) (M1surg), which was shorter than in patients with localized disease (median 20.6 months, 95% CI 16.7–24.6 months) (M0) (*p* = 0.001) (Fig. 1A).

**Table 4:** Univariate and multivariate (*n* = 166) analysis for overall survival

	Univariate analysis	Multivariate analysis		
	*p* value	*p* value	HR	CI (95%)
Tumor location (tail vs head)	0.060	NS	–	–
Age (≥ / < median)	0.002	NS	–	–
Gender (male/female)	0.653	NS	–	–
T-stage (T1, T2/T3, T4)	0.713	NS	–	–
N-stage (N0/N1, N2)	0.295	NS	–	–
M1 (M1/M0)	0.001	NS	–	–
Grading (G1, G2/G3)	0.030	NS	–	–
Pn (Pn1/Pn0)	0.559	NS	–	–
L (L1/L0)	0.606	NS	–	–
V (V1/V0)	< 0.001	< 0.001	2.38	1.54—3.67
R-status	< 0.001	< 0.001	2.29	1.41—3.71
CTx (MD regime vs gemca mono)	0.007	NS	–	–

Univariate analysis was performed by log-rank test

Multivariate analyses were performed by forward logistic regression

Only statistical significant clinicopathological variables are presented

*CI* confidence interval, *CTx* chemotherapy, *HR* hazard ratio, *Pn* perineural invasion, *L* lymphatic invasion, *NS* not significant, *V* venous invasion

^*^p value ≤ 0.05 indicates significance

In multivariate analysis however only positive venous invasion and positive resection margin were left as independent prognostic factors for poor OS (Table 4).

### Overall survival for R0 resected patients

Survival analysis was performed of only R0 resected patients (M0R0 and M1surgR0, *n* = 128, Table 1 and 5). In univariate analysis, patients with PDACs of the pancreatic head, higher median age and positive venous invasion showed a significantly worse prognosis (Table 5). Thus, the median OS with 17.6 months (95 CI 8.8–26.5 months) in patients who received histopathologically proven tumor-free extended resection (M1surgR0, *n* = 17) was not statistically different compared to the median OS with 20.6 months (95% CI 16.7–24.6 months) in patients who received surgery for localized disease (M0, *n* = 131) and to the median OS with 21.1 months (95% CI 17.0–25.2 months) in patients who received histopathological proven tumor-free resection for localized disease (M0R0, *n* = 111) (Fig. 1B, C). In multivariate analysis only positive venous invasion was left as an independent prognostic factor (Table 5).

**Table 5:** Univariate and multivariate (*n* = 128) analysis for overall survival in R0 resected patients

	Univariate analysis	Multivariate analysis		
	*p* value	*p* value	HR	CI (95%)
Tumor location (tail vs head)	0.039	NS	–	–
Age (≥ / < median)	0.006	NS	–	–
Gender (male/female)	0.920	NS	–	–
T-stage (T1, T2/T3, T4)	0.880	NS	–	–
N-stage (N0/N1, N2)	0.693	NS	–	–
M1 (M1/M0)	0.142	NS	–	–
Grading (G1, G2/G3)	0.643	NS	–	–
Pn (Pn1/Pn0)	0.476	NS	–	–
L (L1/L0)	0.779	NS	–	–
V (V1/V0)	0.048	0.010	2.07	1.19–3.58
CTx (MD regime vs gemca mono)	0.058	NS	–	–

Univariate analysis was performed by log-rank test

Multivariate analyses were performed by forward logistic regression

Only statistical significant clinicopathological variables are presented

*CI* confidence interval, *CTx* chemotherapy, *HR* hazard ratio, *Pn* perineural invasion, *L* lymphatic invasion, *NS* not significant, *V* venous invasion

*p value ≤ 0.05 indicates significance

### Overall survival M1surg vs M1pall

**Fig. 2:**
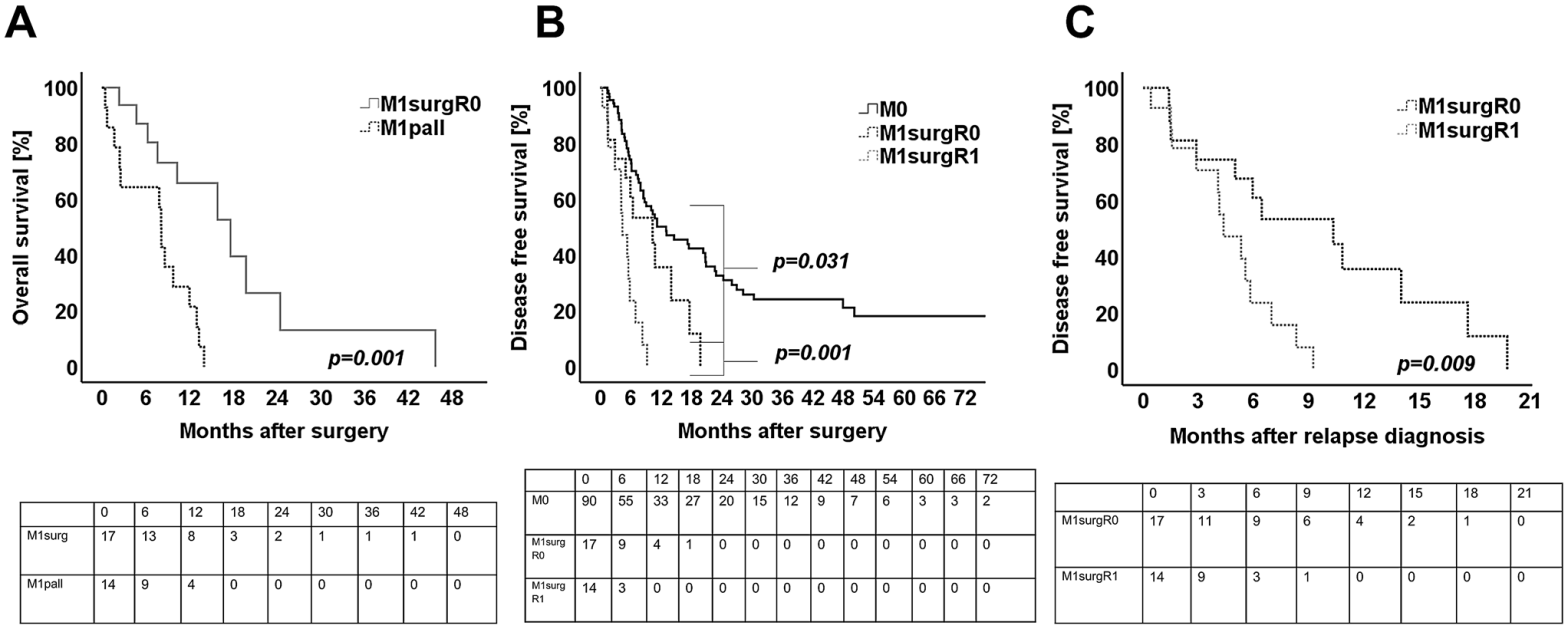
Kaplan Meier survival curves for **A** overall survival of patients after margin-negative extended surgery (M1surgR0, *n* = 17) in correlation to patients after palliative therapy (M1pall, *n* = 14). **B** Disease-free survival of patients without synchronous metastases (M0, *n* = 90) in correlation to patients after margin-negative extended surgery (M1surgR0, *n* = 17, *p* = 0.031) and in correlation to patients after margin positive resections with advanced disease (M1surgR1, *n* = 14, *p* = 0.001) **C** disease free survival of patients after margin-negative resections with synchronous metastases (M1surgR0, *n* = 17) in correlation to patients after margin positive extended surgery (M1surgR1, *n* = 14). Log rank test was used to test for significance. *p* value ≤ 0.05 indicates significance

Survival analysis between M1surg and M1pall patients was performed. In univariate analysis, patients who received extended surgery for metastasized PDACs had a similar survival outcome when compared to M1pall cohort (*p* = 0.051). By considering only margin-negative resected patients for the survival analysis (*n* = 17), patients treated with palliative intent showed a worse survival outcome compared to the M1surg group (*p* = 0.001) (Fig. 2A). No patient after extended resection or palliation (M1surg and M1pall) for oligometastatic disease to the liver was still alive five years after diagnosis.

### Disease-free survival and site of recurrence

Out of the total cohort (*n* = 166, M0 and M1surg) a detailed follow-up of 121 patients was available for disease free survival (DFS) analysis (90 M0 and 31 M1surg). No detailed follow-up information was available for the remaining 45 patients of the study cohort. Anatomic distribution of metachronous disease were summarized in Table 6. While the distribution of metachronous relapse was of statistical significance between group M0 and M1surgR1, site of relapse was homogenously distributed between group M0 and group M1surgR0 (Table 6).

**Table 6:** Distribution of recurrence sites between groups M0, M1 surgR1 and M1 surgR0

	M0			M1surgR1		M1 surgR0	
	*n* = 90		%	*n* = 14	%	*n *= 17	%
No metastases	34		37.8	1	5.6	5	29.4
Hepatic	27		30.0	10	55.6	6	35.3
Pulmonary	9		10.0	3	16.7	4	23.5
Local	18		20.0	0	0.0	2	11.8
Peritoneal	2		2.2	0	0.0	0	0.0
Wilcoxon test		*p* value					
M0 vs. M1surg		0.016					
M0 vs. M1surgR1		0.003					
M0 vs. M1surgR0		0.482					

While the distribution of recurrence sites between groups M0 and M1 surgR1 was heterogeneous, patients after margin-negative resected advanced disease (M1 surgR0) showed a similar distribution to group M0

Wilcoxon test was used to test for statistical significance

*p value ≤ 0.05 indicates significance

At univariate analysis, patients with positive M-status, positive venous invasion and patients with positive resection margins showed a significantly worse DFS when compared to patients after surgery for localized disease (M0) (*p* = 0.031 for M0 vs. M1surgR0 and *p* = 0.001 for M0 vs M1surg R1) (Table 7, Fig. 2B). Thus, the median DFS of 12.9 months (95% CI 6.2–19.8 months) in M0 patients was significantly superior compared to the median DFS of 4.4 months (95% CI 2.3–6.4 months) in M1surgR1 patients and the median DFS of 10.3 months (95% CI 3.3–17.4 months) in M1surgR0 patients (Fig. 2B). When correlating the DFS between group M1surgR1 and M1surgR0, the DFS in patients after extended margin-negative resections (M1surgR0) was significantly prolonged when compared to M1surgR1 (*p* = 0.009, Fig. 2C).

**Table 7:** Univariate and multivariate (*n* = 121) analysis for disease free survival. Univariate analysis was performed by log-Rank test

	Univariate analysis	Multivariate analysis		
	*p* value	*p* value	HR	CI (95%)
Tumor location (tail vs head)	0.052	NS	–	–
Age (≥ / < median)	0.543	NS	–	–
Gender (male/female)	0.739	NS	–	–
T-stage (T1, T2/T3, T4)	0.328	NS	–	–
N-stage (N0/N1, N2)	0.062	NS	–	–
M1 (M1/M0)	< 0.001	0.003	1.556	1.209–2.002
Grading (G1,G2/G3)	0.480	NS	–	–
Pn (Pn1/Pn0)	0.421	NS	–	–
L (L1/L0)	0.612	NS	–	–
V (V1/V0)	0.017	0.032	1.710	1.053–2.776
R-status	< 0.001	0.002	2.057	1.291–3.279
CTx (MD regime vs gemca mono)	0.173	NS	–	–

Multivariate analyses were performed by forward logistic regression

Only statistical significant clinicopathological variables are presented

*CI* confidence interval, *CTx* chemotherapy, *HR* hazard ratio, *Pn* perineural invasion, *L* lymphatic invasion, *NS* not significant, *V* venous invasion

^*^p value ≤ 0.05 indicates significance

At multivariate analysis, only patients with a complete staging including perineural, venous and lymphatic invasion were considered (*n* = 121). Positive M-status, positive venous invasion and positive resection margins were found as independent prognostic factors for DFS (Table 7).

## Discussion

To date, little is known about the feasibility and survival outcome of patients who undergo surgery for synchronously hepatic-metastasized PDACs. To the best of our knowledge, this is the first study to compare survival of patients after extended surgery for synchronous hepatic metastases (M1surg) to patients with localized disease (M0).

Taking the revised eighth edition TNM staging system into account with inclusion of lymphatic, perineural, and venous infiltration, our data demonstrated that patients with isolated synchronous hepatic metastases showed a similar overall survival in multivariate analysis compared to patients with localized disease (group M1surg vs. M0). Length of hospitalization, morbidity and mortality rates did not show any statistical difference between the two groups.

Improved survival outcome by curative surgery, especially in regard to long-term outcome, has never been adequately studied in patients with limited and isolated synchronous hepatic metastases of PDAC. To date, surgery in these cases is not recommended in any current guideline. Curative intended therapy for patients with synchronous hepatic-metastasized colorectal cancer or pancreatic neuroendocrine tumors have been neglected in the past. However, over the last decade surgery became the gold standard of care. Moreover, it has been proven to be oncologically beneficial, to prolong survival, and to improve the quality of life [13, 14]. In PDAC with oligometastatic disease, however, only limited evidence is currently available [15].

It is clear that the decision for a surgical approach is made after subjective reflection of the surgeon. To date, pancreatic resections with synchronous metastasectomies of the liver are rarely performed only in high-volume centers with adequate experience [16]. Thus, to date, only case reports and a limited number of larger case series exist. In previous literature, patients with surgically resected synchronously metastasized PDACs were mostly correlated to patients who were treated in palliative intent [16–20].

In two recent studies, a larger number of patients with synchronously hepatic-metastasized PDACs were analyzed [16, 18]. Six European pancreatic centers retrospectively reported on 69 patients diagnosed with synchronously hepatic-metastasized PDACs, who received simultaneous pancreatic and liver resections [18]. Patients treated in palliative intent served as a control group. A significant benefit for survival was achieved for patients undergoing this extensive surgical approach with tolerable rates of morbidity and mortality compared to patients who only received an exploration (14.5 vs 7.5 months respectively, *p* < 0.001). In a large single-center study from Heidelberg, analogous results were reported [16]. No study compared the survival outcome synchronously oligometastatic resection to patients with localized PDACs (M0). Our results clearly showed for the first time a survival benefit after radical R0 surgery for M1 PDACs with an extended chemotherapy, as survival outcome was similar in patients with localized disease (M0).

Interestingly, the pattern of metachronous metastases was not statistically different in M0 and M1surgR0 patients in our cohort, even if the number of patients included was limited. In both groups, the majority of patients suffered from metachronous hepatic disease. Similar postoperative findings have never been described in previous literature. However, it is known that the foremost primary site of disease recurrence after curative-intended multimodal therapeutic approach for PDAC is the liver [21]. Of note, only patients with complete follow-up were included in the analysis of DFS, resulting in a smaller subset. Yet, as there was no obvious selection bias, our results presumably reflect the statistical relevance of the above mentioned outcomes.

Our study has several limitations including different applied adjuvant treatment regimes. FOLFIRNOX for a multimodal treatment setting was applied in 22.8% of all M1surg and only 8.1% of all M0 patients. An intensified gemcitabine/cisplatin based adjuvant radiochemotherapy was again only administered in M1surg patients. Presumably, this might have influenced the benefit in survival outcome in M1surgR0 patients [9, 22]. Another limitation of this study is that margin-negative resections could not been achieved in ~ 50% of the M1surg patients. The main sites of insufficient margin clearances were after hepatic metastasectomies, presumably due to parenchyma sparing liver resection techniques. Of note, in two out of three M1surg patients who succumbed during the first 30-postoperative days simultaneous hemihepatectomies during pancreatoduodenectomies were performed, which presumably limited the indication window for extended simultaneous hepato-pancreatic surgery in our institution. In our opinion, to secure margin clearance rates and mortality rates in patients who require major hepatic surgery, neoadjuvant therapy in the future will be an obligatory component [9].

The five years survival rate after multimodal therapy for PDAC has not changed over the past decades and is still below 10% [1, 2]. It is therefore not surprising that patients with an initial advanced tumor stage (M1surg) are prone to a less favorable long-term overall survival, presumably due to the high risk of potential development of micro-metastases, especially to the liver. However, due to our findings, we cannot neglect that in a subgroup of patients (R0 resected M1surg and extended adjuvant therapy) a palliative intended therapy would presumably not have shown a similar survival benefit after extended multimodal therapy.

In our opinion, even if patients with synchronously hepatic-metastasized patients are susceptible to micro-metastases, and on the basis of our findings in survival outcome after R0 resection and extended chemotherapy, these new approved chemotherapeutic regimes could help us to open up indication windows for curative-intended therapy. Further multi-centric studies are clearly warranted to analyze the oncological benefit of this interdisciplinary therapeutic approach and foremost the setting of multimodality (neoadjuvant vs. adjuvant) [9, 22, 23]. To our knowledge, similar data is not available in the literature. In our opinion, in a selected group of patients with an excellent ECOG status, a multimodal curative-intended therapeutic approach could be feasible and should not be ignored in the future.

## Conclusion

In summary, selected patients with synchronously hepatic-metastasized PDAC may benefit from extended surgery if an extended chemotherapeutic regime will be applied. Simultaneous pancreatic and liver resections are feasible and well justified by similar morbidity and mortality rates compared to patients with isolated pancreatic surgery. Despite the advanced stage of PDAC, survival outcome after extended surgery was prolonged and thus similar when compared to patients who received surgery for localized PDACs. To validate our results, future studies are warranted to determine which patients may benefit from simultaneous resections [24–26].

## Supplementary Information

Below is the link to the electronic supplementary material.

Supplementary file1 (DOCX 17 KB)

Supplementary file2 (DOCX 17 KB)

## Data Availability

The datasets used and/or analyzed during the current study are available from the corresponding author on reasonable request.

## Abbreviations

Chem: Chemotherapy
CI: Confidence interval
CTx: Chemotherapy
DFS: Disease-free survival
FOLFIRINOX: Folinic acid, fluororuracil, irinotecan, oxaliplatin
Hep: Hepatic
HR: Hazard ratio
Meta: Metachronous/metachronously
OS: Overall survival
Pall: Palliative
PDAC: Pancreatic ductal adenocarcinoma
Pul: Pulmonary
RSS: Relapse specific survival
Surg: Surgical
Syn: Synchronous/synchronously
UICC: Union for international cancer control
