# Supplementary material for: Surgical margin clearance and extended chemotherapy defines survival for synchronous oligometastatic liver lesions of the ductal adenocarcinoma of the pancreas
Source: Int J Clin Oncol. 2021 Jun 16;26(10):1911–21. doi: 10.1007/s10147-021-01961-5 (PMC8449759; doi:10.1007/s10147-021-01961-5)
Supplement: Supplementary file 1 — Supplementary file1 (DOCX 17 KB) [file 10147_2021_1961_MOESM1_ESM.docx]

|  | | | |
| --- | --- | --- | --- |
|  | **M1surg R1**  **n=18** | **M1surg R0**  **n=17** | **Chi-squared test**  ***p-value*** |
|  | **n** | **n** |  |
| **Number of**  **metastases** |  |  | *0.149* |
| Single lesion | 9 | 13 |  |
| 2 lesions | 5 | 3 |  |
| 3 lesions | 3 | 1 |  |
| 4 lesions | 1 | 0 |  |
| **Size of metastases** |  |  | *0.200* |
| <2cm | 12 | 14 |  |
| ≥2cm | 6 | 3 |  |
| **Location of**  **metastases** |  |  | *0.723* |
| left lobe | 9 | 9 |  |
| right lobe | 9 | 8 |  |
| *surg: surgical* | | | |
