# Supplementary material for: Surgical margin clearance and extended chemotherapy defines survival for synchronous oligometastatic liver lesions of the ductal adenocarcinoma of the pancreas
Source: Int J Clin Oncol. 2021 Jun 16;26(10):1911–21. doi: 10.1007/s10147-021-01961-5 (PMC8449759; doi:10.1007/s10147-021-01961-5)
Supplement: Supplementary file 2 — Supplementary file2 (DOCX 17 KB) [file 10147_2021_1961_MOESM2_ESM.docx]

|  |  | | | | |
| --- | --- | --- | --- | --- | --- |
|  | | **M0**  **N=131** | **M1surg R1**  **n=18** | **M1surg R0**  **n=17** | **Chi-squared test**  ***p-value*** |
|  | | **n** | **n** | **n** |  |
| **Chemotherapy** | |  |  |  | ***<0.001*** |
| No CTx | | 11 | 5 | 0 |  |
| Gemcitabine mono | | 80 | 9 | 6 |  |
| Gemcitabine MD | | 35 | 1 | 1 |  |
| FOLFIRINOX | | 5 | 2 | 6 |  |
| HEAT study/RCT | | 0 | 1 | 4 |  |
| *CTx: chemotherapy; Mono: monotherapy; MD: multi-drug; RCT: radiochemotherapy; surg: surgical* | | | | | |
